# Increased Intracranial Pressure Attenuates the Pulsating Component of Cerebral Venous Outflow

**DOI:** 10.1007/s12028-019-00733-4

**Published:** 2019-06-25

**Authors:** Mårten Unnerbäck, Johnny T. Ottesen, Peter Reinstrup

**Affiliations:** 1 Department of Clinical Sciences Lund, Intensive Care and Perioperative Medicine, Lund University, Skåne University Hospital, Malmö, Sweden; 2 Department of Science and Environment, Roskilde University, Roskilde, Denmark; 3 Department of Clinical Sciences Lund, Department of Neurosurgery, Lund University, Skåne University Hospital, Lund, Sweden; 4 IPV SUS Malmö, Inga Marie Nilssons gata 47, 205 02 Malmö, Sweden

**Keywords:** Cerebral blood flow measurement, Cerebral hemodynamics, PC-MRI, Jugular veins, Intracranial pressure

## Abstract

**Background:**

The underlying physiology of the intracranial pressure (ICP) curve morphology is still poorly understood. If this physiology is explained it could be possible to extract clinically relevant information from the ICP curve. The venous outflow from the cranial cavity is pulsatile, and in theory the pulsatile component of venous outflow from the cranial cavity should be attenuated with increasing ICP. In this study, we explored the relationship between ICP and the pulsatility of the venous outflow from the intracranial cavity.

**Methods:**

Thirty-seven neuro-intensive care patients that had been examined with phase-contrast magnetic resonance imaging regarding cerebral blood flow (CBF) through the internal carotid and vertebral arteries and venous flow in the internal jugular veins were retrospectively included. The pulsatility of the jugular flow was determined by calculating the venous pulsatile index. The results were correlated to clinical data registered in the patient data monitoring system, including ICP and cerebral perfusion pressure (CPP).

**Results:**

CBF was 996 ± 298 ml/min, and the flow in the internal jugular veins equaled 67 ± 17% of the CBF, with a range of 22–97%. The venous pulsatile index correlated negatively to ICP (*R* = − 0.47 *p* = 0.003). The lowest flow in the internal jugular veins over the cardiac cycle (*F*_min_) was not correlated to ICP. Temperature, end-tidal CO_2_, MAP, and CPP were not correlated to venous pulsatility.

**Conclusion:**

An increase in ICP correlates to a lower pulsatility of the venous outflow from the cranial cavity. A lower pulsatility could be due to increased pressure requirements to compress intracranial veins with increasing ICP.

## Introduction

Generation of intracranial pressure (ICP) is dependent on the rigid cranium enclosing the cranial cavity. The content of the cavity is composed by the brain, venous- and arterial blood, and the cerebrospinal fluid (CSF), all of which are virtually incompressible. The Monro–Kellie doctrine states that any expansion of one of these components must be compensated by a displacement of volume from one or more of the others [1]. The cerebral blood flow (CBF) varies over the cardiac cycle. The peak during systole must be compensated by an extrusion of CSF and especially venous blood. A plausible explanation is that the ICP increase due to arterial influx drives the displacement of venous blood by compression of the thin-walled veins, creating pulsations of the venous outflow [1–5].

The physiology causing the ICP curve morphology is still unclear [6, 7]. Early studies suggested that both arterial blood pressure and central venous pressure pulsations could affect the ICP curve morphology [8–10]. The intracranial hydrodynamics are strongly connected to the ICP curve and highly dependent on the venous outflow from the cranial cavity [11]. A recent study using magnetic resonance imaging (MRI) phase-contrast technology has presented that the flow of arterial and venous blood as well as the displacement of CSF within the central nervous system could explain the shape of the ICP curve [5]. However, the thin-walled venous segments are subject to a general compression from an increase in mean ICP, reducing the cerebral venous blood pool. This reduction in the venous bed should increase flow velocity in the venous system, attenuating the pulsatile component of the venous outflow from the intracranial cavity due to the cyclic rise in ICP [12].

The flow in arteries and veins may be measured using phase-contrast MRI [13]. Summarizing the flow in the carotid and vertebral arteries has been used to calculate CBF [14], and the venous outflow from the cranial cavity can be measured in the internal jugular veins [15].

To test the hypothesis that increased mean ICP reduces the pulsatile part of the cerebral venous flow, we used MRI cine phase-contrast examinations and ICP measurements from neuro-intensive care patients.

## Materials and Methods

### Inclusion Criteria

Ethical approval for the study was granted by the Regional Ethical Review Board at Lund University (2014/403). The database of patients monitored regarding ICP with an intraventricular device and examined with phase-contrast MRI at the neuro-intensive care unit at Skåne University Hospital during the period 2008–2017 was searched. All examinations were evaluated regarding quality, and patients with examinations sufficient to measure blood flow in the internal carotid, vertebral arteries, and venous flow in the internal jugular veins were included. All cases with a non-intact cranial cavity, with the exception of the insertion hole of the ventricular catheter, were excluded.

### MRI Examinations

The MRI examinations were performed using Philips Intera 1.5 T or Philips Intera 3.0 T. The patients were placed supine during the examination. A velocity-encoded cine phase-contrast pulse sequence using Electro cardiogram (ECG) gating was used. Measurements were made in slices, placed just under the foramen magnum perpendicular to the vessels. The level of the through plane was chosen on a two-dimensional lateral angiographic image of the neck. The slices were 6 mm thick, and a 256 × 128 matrix had been used. Field of view was 18 cm. Velocity encoding value had been set to 90 cm/s. TR was 26 ms, and the flip angle was 15°. Each cardiac cycle was sampled at 30–35 time points.

**Fig. 1:**
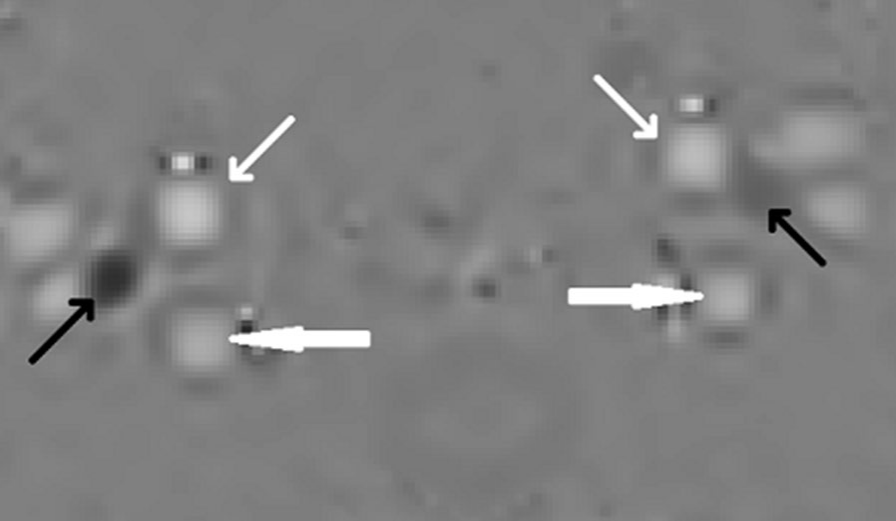
The arteries and veins used for measurements of cerebral blood flow and flow in the internal jugular veins. The small white arrows point at the internal carotid arteries, the large white arrows point at the vertebral arteries, and the black arrows point at the internal jugular veins. White color represents flow into the cranial cavity, and black color represents flow out from the cranial cavity. The intensity of the color represents the flow value. Note the difference in both size and flow between the left and the right internal jugular veins

All MRI examinations were analyzed using the freely available software SEGMENT v 2.0 R5432 [16]. One examiner performed the analysis of the MRI examinations. A region of interest was drawn manually over each of the vessels in the phase image with the maximum flow. The flow was acquired by the software pixel by pixel with a temporal resolution of 30–35 slices per cardiac cycle. The same segmentation was used over the entire cardiac cycle (Fig. 1).

**Fig. 2:**
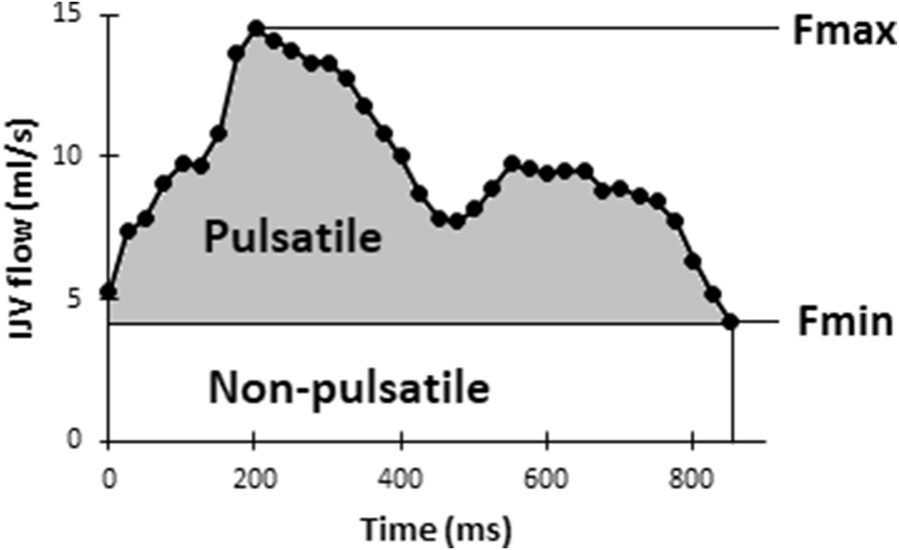
The flow rates over one cardiac cycle in one individual. IJVflow, the combined flow in the internal jugular veins, *F*_max_, maximum flow, *F*_min_ minimum flow. The pulsatile component of internal jugular vein flow is represented by the gray area; the non-pulsatile component of internal jugular vein flow is represented by the white area

The CBF was calculated by summarizing the flows in the carotid and vertebral arteries. The internal jugular vein flows were evaluated in the same manner and summarized providing the internal jugular venous flow. The internal jugular vein flow ratio, i.e., the part of the cerebral venous outflow flowing through the internal jugular veins, was calculated by dividing internal jugular venous flow with CBF. The venous pulsatile index was used to correlate venous pulsatility to ICP. Venous pulsatile index is calculated by the formula 100 × (*F*_max_ − *F*_min_)/*F*_max_, where *F*_max_ is the maximum flow and *F*_min_ is the minimum flow over the cardiac cycle [15] (Fig. 2).

### ICP Measurements

ICP was monitored invasively with a 8F tunneled intraventricular catheter (HanniKath, Smiths Medical Deutschland GmbH), inserted through a burr hole. The catheter was connected to a CSF drainage set with a microtransducer (HanniSet, Smiths Medical Deutschland GmbH). The transducer was zeroed against atmospheric pressure at the highest point of the cranium and connected to a Philips Intellivue MP70 STAD. The data were automatically registered in the patient data monitoring system (PDMS) (IntelliSpace Critical Care and Anesthesia, Philips). The last mean ICP registered before the MRI examination was extracted from the database. Patients were positioned in a 30° head up position.

### Clinical Data

Clinical data were retrieved from the PDMS, including diagnosis, mean arterial blood pressure (MAP), end-tidal CO_2_, and temperature. The last measurements before the MRI examination were used. The existence of a central venous access and its location was noted.

### Statistical Analysis

For statistical analysis, IBM SPSS Statistics for Windows, version 22.0. (IBM Corp, USA) was used. Linear regression was used to test correlation. The Mann–Whitney *U* test was used to test the null hypothesis between samples, and a Chi-square test was used in case of dichotomous variables. All values are given as mean with standard deviation, unless otherwise stated.

## Results

A total of 37 patients had complete datasets and could be included. The diagnosis was traumatic brain injury (TBI) in 15, subarachnoid hemorrhage (SAH) in 11, meningitis in 5, intracerebral hemorrhage in 2, hydrocephalus in 2, multiple arterial emboli in 1, and meningioma in 1.

The mean age of included patients was 50 ± 14 years. ICP was 9 ± 7 mmHg and cerebral perfusion pressure (CPP) 80 ± 14 mmHg. Age was significantly lower in the TBI patients compared to SAH (*p* = 0.01). There was no significant difference between the two major diagnosis groups TBI and SAH regarding ICP and CPP (ICP *p* = 0.89, CPP *p* = 0.09).

CBF was 996 ± 298 ml/min with no significant difference between TBI and SAH, and the internal jugular vein flow ratio equaled 67 ± 17% of the CBF, with a range of 22–97%. Six individuals had a flow through the internal jugular veins which constituted less than 50% of CBF.

Venous pulsatile index was 55 ± 15. The maximum internal jugular vein flow rate (*F*_max_) was 1.78 ± 2.50 times the lowest flow (*F*_min_) over the cardiac cycle. There was no difference in the venous pulsatile index between individuals with a venous drainage through the internal jugular veins of > 50% compared to < 50% (*p* = 0.84). Neither the internal jugular vein flow ratio nor the internal jugular vein flow was correlated to ICP (internal jugular vein flow ratio *p* = 0.75 and internal jugular vein flow *p* = 0.93). No significant difference between TBI and SAH patients regarding these parameters was found (CBF *p* = 0.93, internal jugular vein flow ratio *p* = 0.97 and venous pulsatile index *p* = 0.34) (Table 1).

**Table 1:** Clinical data and flow measurements divided into diagnosis groups

	TBI	SAH	Meningitis	Other^a^	All individuals
*n*	15	11	5	6	37
Age (years)	44 ± 14	57 ± 8	52 ± 10	54 ± 16	50 ± 14
Sex (M/F)	14 M/1 F	4 M/7 F	3 M/2 F	3 M/3 F	24 M/13 F
ICPmean (mmHg)	8 ± 9	9 ± 7	11 ± 11	10 ± 7	9 ± 7
MAP (mmHg)	84 ± 11	94 ± 14	90 ± 14	92 ± 6	89 ± 13
CPP (mmHg)	75 ± 12	85 ± 15	79 ± 17	82 ± 11	80 ± 14
CBF (ml/min)	981 ± 295	990 ± 224	1035 ± 271	1014 ± 419	996 ± 298
IJVflow (ml/min)	665 ± 233	671 ± 256	541 ± 228	812 ± 388	674 ± 280
IJVarea (cm^2^)	0.90 ± 0.44	1.13 ± 0.52	1.18 ± 0.71	1.02 ± 0.46	1.02 ± 0.52
VPI	52 ± 16	59 ± 13	58 ± 19	52 ± 10	55 ± 15

Data are given in mean ± SEM

*CBF* cerebral blood flow, *CPP* cerebral perfusion pressure, *ICPmean* mean intracranial pressure, *IJVarea* the combined area of the internal jugular veins, *IJVflow* flow through the internal jugular veins, *MAP* mean arterial pressure, *SAH* subarachnoid hemorrhage, *TBI* traumatic brain injury, *VPI* venous pulsatile index

^a^Other include one multiple cerebral arterial emboli, two intracerebral hemorrhage, two hydrocephalus and one meningioma

Eight individuals had flow in only one internal jugular vein, six in the left and two in the right. Fifty-one percent of the individuals had a dominant flow in the right internal jugular vein. A total of 20 patients had an indwelling central venous catheter during the MRI examination. Sixteen had the central venous catheter placed in the right internal jugular vein, one individual had the central venous catheter placed in the right external jugular vein, one in the right subclavian vein, and two individuals had a central line in the left internal jugular vein. Comparing the group with a central line placed in the right internal jugular vein to those without a central line in the right internal jugular vein, there was no significant difference regarding dominant flow side or internal jugular venous flow ratio (dominant flow side *p* = 0.25 and internal jugular venous flow ratio *p* = 0.37).

**Fig. 3:**
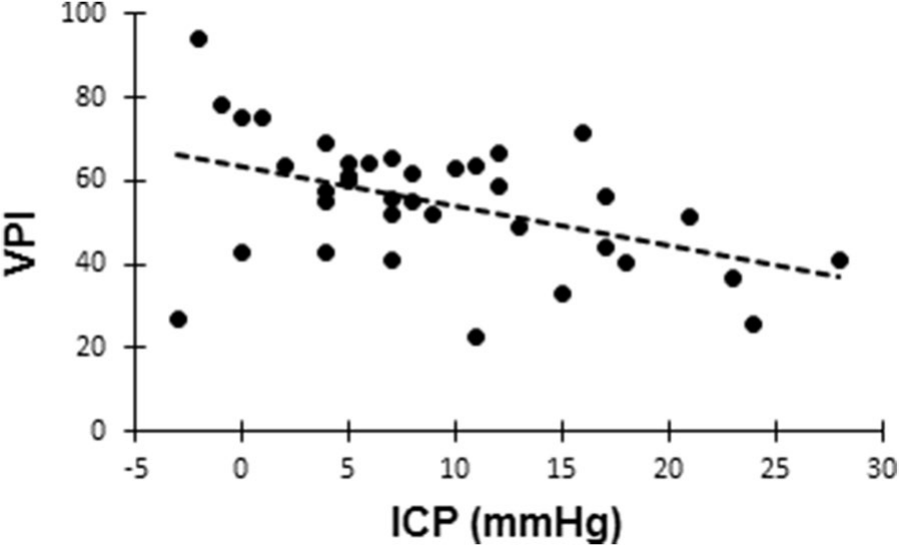
Regression plot of the venous pulsatile index (VPI) against intracranial pressure (ICP). *R* = − 0.47, *p* = 0.003

The venous pulsatile index correlated negatively to an increase in ICP (*R* = 0.47 *p* = 0.003) (Fig. 3). There was no correlation between CPP and venous pulsatile index (*R* = 0.17 *p* = 0.32) No correlation was found between ICP and *F*_min_. (*R* = 0.15 *p* = 0.37). Temperature, end-tidal CO_2_, and MAP were not correlated to venous pulsatile index.

## Discussion

**Fig. 4:**
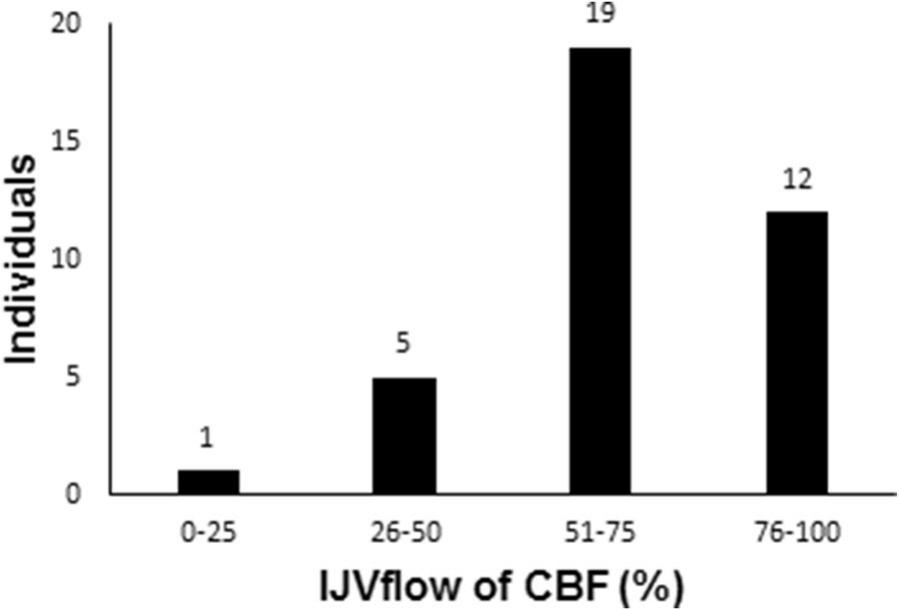
Distribution of the amount of cerebral blood flow (CBF) drained through the internal jugular veins (IJV_flow_). Number of individuals plotted against percentage of CBF flowing through the internal jugular vein (IJV)

The venous outflow from the cranial compartment may take several routes [15, 17]. In the data presented in this study, the amount of venous blood flowing through the internal jugular veins compared to CBF was in the range 22–97%, with a mean value of 67% (Fig. 4). The outflow through the internal jugular veins is not evenly distributed, and most had a dominant flow in one internal jugular vein and 5% with no flow in one of the internal jugular veins. Both findings were in accordance with previous findings in healthy volunteers investigated with ultrasound technique and phase-contrast MRI [17, 18].

Independent of the route the blood leaves the cranial cavity venous outflow was early suspected of influencing the ICP [10], and the venous outflow affects the intracranial hydrodynamics and the ICP [11]. Several studies have shown that resistance to cerebral venous outflow increases ICP [19–21] and furthermore suggested that the cerebral venous outflow affects the single ICP curve form in conjunction with CBF and the CSF flow over the foramen magnum [3, 22]. Recently, there has emerged further evidence in support of this [5]. In this study, we set out to find evidence of the relationship between mean ICP and cerebral venous flow. With rising ICP due to mass lesion, brain swelling, or hydrocephalus, the intracranial venous blood pool has to be reduced due to the physiological properties of the intracranial cavity in accordance with the Monro–Kellie doctrine [1]. To this end, the increase in baseline ICP will compress the thin-walled intracranial veins and the pressure inside the veins will equal the ICP. Since the capillary flow is almost non-pulsatile [23], the veins are constantly filled with blood from the capillary network, which must be expelled to the sinuses. As the vein’s diameter are reduced, and if CBF is unchanged, the total venous flow velocity must be increased, and even more pressure is required to compress the veins further. The lowest flow over the cardiac cycle then has to increase in order to accommodate the venous flow, and the increased intravenous pressure counteracts the compression of the veins by the increased ICP during systole. As a result, an increased mean ICP would cause venous outflow to variate less over the cardiac cycle as found in our study. Transcranial Doppler ultrasonography (TCD) has been used to demonstrate a correlation between a rise in mean ICP and a rise in mean blood flow velocity in the basal vein of Rosenthal, but with no correlation between ICP and pulsatility index [24]. These data must, however, be interpreted with the understanding that pulsatility index is calculated using flow velocities and not volumes [25].

Phase-contrast MRI was used to measure flow [13]. The technique is well established and has been used to measure both CBF and flow in the internal jugular veins in several studies [2–5, 12, 14, 15, 26–28]. In a study using a variant of phase-contrast MRI, it was possible to assess the respiratory influence on internal jugular vein pulsatility [29]. The technique used in this study samples the flow over several respiratory cycles, canceling the cyclic effect of respiration on central venous flow. In studies, where venous outflow has been put into relation with arterial inflow, it has been suggested prudent to correct venous outflow data by multiplying it with a correction factor to equal arterial inflow [2]. However, the venous pulsatile index takes into account the maximum and minimum flow and their relation, with a higher venous pulsatile index in the presence of a more pulsatile flow [15]. Using venous pulsatile index, a correction of the flow in the internal jugular vein is not necessary, since the results are independent of such a correction. In contrast, a comparison between minimal flow and ICP requires a correction since it is absolute and dependent on the amount of total venous outflow through the internal jugular vein.

In the data presented in this study, there is a correlation between mean ICP and the degree of pulsatility of the venous outflow over the cardiac cycle using venous pulsatile index. Inter-individual variation should be expected, since the physiological state of each patient differs. The correlation was significant, though variations could be observed (Fig. 3). This supports the theory that increased mean ICP would lead to a less pulsatile flow within the mean ICP span investigated in this study. Higher mean ICP levels may create quite different venous flow variations.

A rise in mean ICP lowers the pulsatile component of the venous outflow, and in order to have an unchanged venous outflow the non-pulsatile component must increase (Fig. 2). We did not find a correlation between increasing mean ICP and increased *F*_min_, although a correlation between minimal flow velocity and ICP has previously been demonstrated using TCD [24]. However, the *F*_min_ is an absolute value and thereby highly dependent on the ratio of the cerebral venous outflow flowing through the internal jugular veins. With just small variations in the *F*_min_ together with the high level of inter-individual variation regarding the internal jugular venous ratio of cerebral venous outflow, such a correlation could easily be missed.

The venous pulsatile index was generally higher in our study compared to a previous study in healthy volunteers [15]. This could be due to methodical differences as the CBF in this study was higher than earlier reported [2, 14, 26–28], but also the fact that the previous study included the epidural vein. The pulsatile flow in the epidural vein, at the foramen Monro level, should be smaller as the vein does not leave the central nervous system and therefore not subjected to the same difference in outflow pressure.

Although the scope of this study was to examine the interaction between venous outflow and mean ICP, the data regarding CBF must be addressed. Previous research using healthy volunteers and patients with stable diseases has reported CBF values in the range of 657–825 ml/min [2, 14, 26–28]. The population in this study has a significantly higher CBF, 996 ml/min, although with a high degree of variation. All subjects included in this study required neuro-intensive care and were treated at the neuro-intensive care department in Lund. Measuring CBF with phase-contrast MRI in this type of patients has, to our knowledge, not been used clinically, and no data regarding this kind of patients have, to our knowledge, been reported previously. All patients had been examined in a relative stable phase, and none of the SAH patients was suspected to have intracranial arterial vasospasm. Meningitis has been shown to induce hyperemia [30], but the increased CBF in TBI and SAH patients was more surprising. The elevated CBF found in our study could be due to a relative loss of cerebral arterial autoregulation. The impact of this feature in patients with TBI and its implication on outcome has been the focus for research during the past years [31]. The high CBF could possibly explain the surprisingly high venous pulsatile index detected in our material, since a high pulsatile arterial inflow compressing the veins could be expected in this situation.

In a theoretical model where ICP over the cardiac cycle mainly is influenced by the inflow of arterial blood, the venous outflow, and the oscillating movement of CSF over the foramen magnum, venous outflow becomes a more passive component, driven by the inflow of arterial blood [1]. How the venous outflow is affected in different physiological states would directly affect the morphology of the ICP curve [11]. In order to understand the physiology and understand the ICP curve, it is important to understand how the venous outflow is affected by the surrounding parameters. We do believe that this study supports the theory that increasing mean ICP, within this range, reduces the intracranial venous blood pool and thereby compromises the intracranial cavity’s ability to cope with the rapid influx of arterial blood modifying the ICP curve form. With increasing mean ICP, less venous blood is displaced due to the ICP amplitude, causing a less pulsating venous flow, as observed in this study.

## Conclusion

In conclusion, the main finding of our study suggests that the flow patterns of the venous outflow from the cranial cavity are correlated to ICP, where an increase in ICP is associated with a smaller pulsating component. This finding is important since it could affect the cranial cavity’s ability to handle the rapid influx of arterial blood during systole and thus cause changes in ICP curve morphology.

## Abbreviations

CBF: Cerebral blood low
CPP: Cerebral perfusion pressure
MAP: Mean arterial pressure
SAH: Subarachnoid hemorrhage
TBI: Traumatic brain injury
VPI: Venous pulsatile index
